# Refractory burning mouth syndrome: clinical and paraclinical evaluation, comorbidities, treatment and outcome

**DOI:** 10.1186/s10194-017-0745-y

**Published:** 2017-03-29

**Authors:** Dimos D. Mitsikostas, Srdjan Ljubisavljevic, Christina I. Deligianni

**Affiliations:** 1 Aeginition Hospital, National & Kapodistrian University of Athens, Athens, Greece; 2 Clinical Center of Nis, Clinic for Neurology, University of Nis, Nis, Serbia; 3 Neurology Department, Athens Naval Hospital, Athens, Greece

**Keywords:** Burning mouth syndrome, Clinical picture, Treatment, Venlafaxine, Clonazepam, Outcome

## Abstract

**Background:**

Burning Mouth Syndrome (BMS) is a chronic pain condition characterized by persistent intraoral burning without related objective findings and unknown etiology that affects elderly females mostly. There is no satisfactory treatment for BMS. We aimed to observe the long-term efficacy of high velanfaxine doses combined with systemic and topical administered clonazepam in a particular subgroup of BMS patients who do not respond to current clinical management.

**Results:**

Eight (66.1 ± 6.2 years old females) out of 14 BMS patients fulfilled the inclusion criteria and were treated with venlafaxine (300 mg/d) and clonazepam (5 mg/d) for 35.4 ± 12.1 (mean ± SD) months. The average duration of the symptoms at baseline was 4.3 ± 1.4 years and the overall mean daily pain intensity score was 8.6 ± 1.3 (VAS); pain was in tongue and within the oral mucosa, accompanying by oral and facial dysesthesia. In five patients tasting was abnormal. All patients had positive history of concomitant primary headache. The average score of Hamilton Rating scale for Anxiety and Depression was 21 ± 4.2, and 26.1 ± 2.9, respectively. Previous ineffective treatments include anticonvulsants and anti-depressants. All patients responded (more than 50% decrease in VAS) after three months treatment (mean VAS 3.2 ± 2.2) with no remarkable adverse events.

**Conclusion:**

BMS deserves bottomless psychiatric evaluation and management when current available treatments fail. Treatment with venlafaxine combined with topical and systemic clonazepam may be effective in refractory BMS cases but further investigation in a large-scale controlled study is needed to confirm these results.

## Background

BMS is a chronic pain condition characterized by persistent intraoral burning without related objective findings and unknown etiology that predominantly affects elderly females. Previously used terms include stomatodynia, or glossodynia when confined to the tongue (based on the Greek words stoma that means mouth and glossa that means tongue). The condition has been recently recognized in the current International Classification for Headache Disorders (ICHD-III beta) as an intraoral burning or dysaesthetic sensation, recurring daily for more than 2 h per day over more than 3 months, without clinically evident causative lesions. Oral mucosa should appear normal with normal sensory testing [1]. How prevalent the condition is remains debatable but recent general population based studies showed an incidence of 11.4 per 100,000 person-years [2] and a point prevalence of 0.11%, or 105.6 (95% CI, 88.6–122.6) per 100,000 persons [3]. Age-adjusted prevalence in women was significantly higher than men: 168.6 (95% CI, 139.0–198.2) vs. 35.9 (95% CI, 21.4–50.3) per 100,000 persons (*P* < 0.001). The highest prevalence was in women aged 70 through 79 years (527.9 per 100,000 persons). Mean (SD) age at BMS diagnosis was 59.4 (15.1) years (range, 25–90 years) [3]. In other reports the BMS prevalence varies from 3.7% up to 40% in elderly [4]. Little is known regarding the aetiopathology of the condition. There is evidence that BMS is related to mild sensory and autonomic small fiber neuropathy with concomitant central disorders particular within the brainstem [5, 6], as well as to impaired endogenous dopamine system in the putamen [7]. In other reports MBS is often comorbid with mood and somatoform disorders with prolong immunoendocrine changes [8]. In some refractory BMS cases comorbided mood disorders are severe [9, 10]. Altered structure and function in the hippocampus and medial prefrontal cortex in BMS patients explain the concomitant affective conditions [11].

There is no satisfactory treatment for BMS. The most widely accepted treatment options that show variable results include tricyclic anti-depressants, benzodiazepines, antipsychotic drugs [12], and SSRIs [13]. Because there is enough evidence that in BMS patients TRPV1 receptors are over-expressed [14] local application of capsaicin was used efficiently [15]. The best evidence for BMS treatment includes only topical capsaicin, alpha-lipoic acid (ALA), and clonazepam [16, 17]. Interestingly, there are reports of BMS comorbidity with Parkinson disease (PD) or Restless Syndrome [18–20] and dopamine agonists showed potential efficacy in reducing BMS symptoms [21, 22]. Non-pharmaceutical treatments may help in addition, like cognitive psychotherapy [23]. Nevertheless, outcome for BMS is poor. In a study of 91 BMS patients the symptoms remitted spontaneously within the five years of treatment. Only 42% of the study population had improved the symptomatology significantly, and this improvement would reach 60% if clonazepam were associated to psychotherapy [24].

In this study we aimed to observe the long-term efficacy of high velanfaxine doses combined with clonazepam in a particular subgroup of BMS patients who do not respond to current clinical management.

## Methods

Patients with burning mouth symptoms that were resistant to current available treatments were selected at presentations for long follow-up, since the beginning of 2010. After the publication of ICHD-III beta all patients fulfilled the BMS criteria were identified and among them those they had no improvement after at least three consecutive drug treatments were followed. Pharmacotherapy might include any agent for chronic pain alone, or in combination with psychotherapy, counting anti-depressants and anti-epileptics in various doses and treatment durations, without any specific pattern. After three consecutive treatment attempts without a 50% reduction in daily pain, as defined by patients them selves, the selection criterion was fulfilled. No exclusion criteria were applied. Once a patient had recruited, a daily pain diary was delivered to follow pain duration and severity during follow-up period. A relevant physician treated any concomitant non-neurological condition according to current clinical practice but treatments with local anesthetics, anti-epileptics and anti-depressants were not allowed. Antiarrhythmic agents that may have analgesic effects could be used only after approval by the research team. All participants were told that they will get two treatments simultaneously that have been trialed alone, but not in combination before. The potential treatment adverse effects were announced as well. A specific investigation protocol was designed for diagnostic reasons including brain and facial MRI, trigeminal blink reflex and peripheral nerve conduction studies apart from other tests necessary because of patients’ particular comorbidity and medical history. Participants allowed to performed neurophysiological studies of facial nerve in private laboratories, thus no common methodology used. All patients had a specific oral and orofacial clinical examination to exclude any oral condition or temporomandibular joint and occlusion dysfunction from an expert. In addition all patients had a neuropsychological evaluation with a battery of tests including the Hamilton Rating Scales for Anxiety and Depression, as well as clinical psychiatric consultation. In all participants cognitive psychotherapy treatment was suggested, in addition to pharmacotherapy. Participants used the analogue visual scale (VAS) to assess mean daily pain.

In all patients venlafaxine was added at high doses (300 mg daily), together with clonazepam. Oral solution of clonazepam was used (Rivortil**®** oral solution 2.5 mg/ml). Patients were asked to keep the solution in their mouth for 5 min before swallowing, in one evening dose of 2.5 mg. After 5 days the dose increased up to 5 mg daily, divided into three doses (1.25 mg in the morning, 1.25 in the afternoon and 2.5 mg at bedtime). Always the solution should be kept in the mouth for at least five minutes before ingestion. Previous treatments were removed gradually within 6 months in six cases.

All participants singed inform consent. The Ethical and Scientific Committee of the Athens Naval Hospital approved the study.

## Results

Eight females out of 14 patients with refractory BMS cases were included in the protocol. All were above 58 years old (mean ± SD: 66.1 ± 6.2) with the mean duration of the condition lasting 4.3 ± 1.4 years. All were followed-up in our department for more than 2 years (mean follow-up duration 35.4 ± 12.1 months).

### Clinical picture and comorbidity

The clinical characteristics of patients are presented in Table 1. Typically, pain was severe (mean VAS score 8.6 ± 1.3) and neuralgiform (resembling neuralgia) in all cases but strictly unilateral only in three cases, located within the tongue and the additional oral mucosa. In all cases pain was triggered or exaggerated by several stimuli including mastication, tongue movements, altered food temperature and six patients had lost wait significantly. Pain was daily, lasting for more than two hours and was typically exaggerated during the bedtime. Accompanying local symptoms included mouth dryness, altered taste and oral dysesthesia. No facial autonomic symptoms patients reported. All reported previous headaches classified retrospectively by the authors according to the ICHD-IIIbeta [1] as migraine in six cases and tension-type headache in two cases. But during examination and follow-up none reported concomitant headaches, apart from unspecified lightheadedness. The most common general symptoms were fatigue and insomnia, present in all cases. According to Hamilton rating scale for Depression (HAM-D) all showed several depressive symptoms (mean HAM-D26.1 ± 2.9 and for anxiety 21.0 ± 4.2) and all underwent a psychiatric consultation that revealed depression, or dysthymia. In five cases depression was already mentioned in their medical records and has been treated with several anti-depressants previously. In addition some cases were also diagnosed with general anxiety disorder, or somatoform disorder, or panic attacks by psychiatrists. The most common additional concomitant medical condition was arterial hypertension, followed by Parkinson disease (PD) and hypothyroidism (Table 1). No patient needed antiarrhythmic agents. Notably, only three patients had the BMS diagnosis previously. Three patients had no diagnosis and two had the diagnosis of atypical orofacial pain.

**Table 1:** Clinical characteristics of patients

Case	1	2	3	4	5	6	7	8	Mean (±SD)
Age at presentation	60	58	62	68	71	73	63	74	66.1 (6.2)
Mean pain severity (0–10)	7	8	7	9	8	6	7	9	7.6 (1.2)
Disease duration since first symptom (years)	3.5	2	3	5	4	5.5	6.5	5	4.3 (1.4)
Pain location in tongue	Bil.	Un.	Bil.	Un.	Bil.	Bil.	Un.	Bil.	
Pain location in additional oral mucosa	Yes	Yes	No	Yes	Yes	Yes	No	Yes	
Pain duration per day in hours	>8	>6	>6	3–8	2–4	4–8	>6	>10	>2
Pain during night	Yes	Yes	Yes	Yes	Yes	Yes	Yes	Yes	
Mouth dryness	Yes	No	Yes	No	Yes	Yes	No	Yes	
Altered taste	Yes	No	Yes	No	Yes	Yes	No	Yes	
Oral dysaesthesia	Yes	Yes	Yes	Yes	Yes	Yes	Yes	Yes	
Facial dysaesthesia	No	No	No	Yes	No	No	No	Yes	
Follow-up (months)	27	16	37	48	52	34	26	43	35.4 (12.1)
Number of previous treatments	3	4	5	4	6	4	8	5	4.9 (1.5)
Comorbidity	HY, HT	OB, HY	DP, OB	HY, PD, DP	HT, DP	CH, CHD	HT, DP, PD	DP, HY	
History or concomitant primary headache	Yes	Yes	Yes	Yes	Yes	Yes	Yes	Yes	
HAM-A	17	16	20	17	24	28	24	22	21 (4.2)
HAM-D	21	24	25	25	30	29	28	27	26.1 (2.9)
Taste tests	Abnormal/Bi	Normal	Abnormal/Bi	Normal	Abnormal/Bi	Abnormal/Bi	Normal	Abnormal/Bi	

*Bil.* Bilateral, *Un.* Unilateral, *HY* Hypertension, *HT* Hypothyroidism, *OB* Obesity, *DP* Depression, *PD* Parkinson Disease, *CHD* Coronary Heart Disease, *HAM-A* Hamilton rating scale for anxiety, *HAM-D* Hamilton rating scale for Depression. Number of previous treatments refers to drugs the patients had given for Burning Mouth Syndrome specifically and not for any other comorbid condition

Neurological and physical examination were normal in all cases apart from those they co-suffered from PD (only mild extrapyramidal symptoms and sings; all three cases were l-dopa responders to mild doses). Sensory tests in face for small and large fibers were within normal limits in all patients (no distal loss of pinprick or thermal sensation, nor punctate hyperalgesia or brush evoked allodynia were observed). Bilateral tongue taste tests (using salty, bitter, sour or sweet solutions) were abnormal however in five cases (patients also reported changes in taste). Testing was unpleasant in all cases because it triggered pain.

### Paraclinical investigation

Brain and facial MRI was performed in all cases. In five patients medium small vessel disease was found in brain with periventricular location. In one patient lacunes with midbrain were seen in addition. Facial MRIs showed no abnormalities related to BMS clinical picture. Facial nerve conduction studies were normal in all cases (velocity was within the normal values adjusted for patients’ age). In three cases ipsilateral to pain a slight decrease in facial nerve conduction was observed (still within the normal values) and blink reflexes were performed that were normal (R1 and R2 responses) without significant differences between two sites (patient with midbrain lacunas included). Thus, in all cases paraclinical investigation, including routine blood tests (CRP, leucocytes) did not reveal any significant abnormality, related to patients’ symptoms.

### Treatment and outcome

Previous treatments included anticonvulsants (pregabaline, gabapentine, topiramate, carbamazepine, lamotrigine and phenyntoin) and anti-depressants (amitriptyline, venlafaxine, mirtazapine and duloxetine) in various doses (Table 2). No patient received treatment with a topical anesthetic during the observation treatment period.

**Table 2:** Previous treatments that failed to improve BMS

Case 1	Pregabaline 150 mg/d plus topiramate 200 mg/d for 5 months; carbamazepine 800 mg/d for 3 months
Case 2	Pregabaline 75 mg/d for 3 months; amitriptyline 75 mg/d for 6 months; phenyntoin 300 mg/d for 2 months
Case 3	Gabapentine up to 2.400 mg/d for 3 months; amitriptyline 75 mg/d plus carbamazepine 400 mg/d for 3 months
Case 4	Amitriptyline 100 mg/d for 6 months; gabapentine 1.800 mg/d plus duloxetine 60 mg/d for 3 months
Case 5	Pregabaline 300 mg/d plus duloxetine 60 mg/d for 3 months; carbamazepine 600 mg/d for 3 months
Case 6	Venlafaxine 150 mg/d plus gabapentine 1.600 mg/d for 3 months; amitriptyline 75 mg/d plus topiramate 200 mg/d for 3 months
Case 7	Pregabaline 150 m/d plus amitriptyline 75 mg/d for 1 month; carbamazepine 800 mg/d for 1 month
Case 8	Carbamazepine 800 mg/d plus mirtazapine 30 mg/d for 3 months; douloxetine 90 mg/d plus lamotrigine 200 mg/d for 3 months

**Fig. 1:**
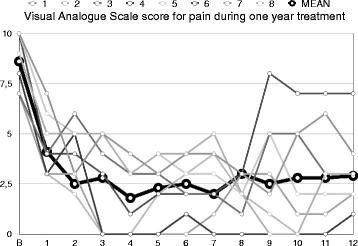
Mean Visual Analog Scale scores per month for eight patients during the first year of treatment. Bold line represents the men of eight scores

In cases 3 and 8 (Table 2) gabapentin 600 mg/day and carbamazepine 400 mg/day were continued because pain relapsed after withdrawal. Only three patients accepted to perform psychotherapy (cases 2, 3 and 7). All eight patients responded (more than 50% decrease in pain intensity daily) after three months treatment (mean VAS 2.8 ± 2.2). Three out of eight patients became pain free and two reported more than 75% decrease of pain severity, but within the entire period of follow-up, almost all patients relapsed. Relapses were treated successfully by adding 1.25–2.5 mg/day of clonazepam. For one pain-free-patient who was not relapsed treatment was discontinued after one year; she regressed and treatment restarted over again (Fig. 1). Treatment adverse events included sedation (in 5 out of 8 patients), dry mouth (8/8), constipation (6/8), increase in blood pressure (1/8), drowsiness (8/8), fatigue (3/8) and irritability (1/8). By time these adverse events were tolerated remarkably. Two cases were overused clonazepam (up to 10 mg per day) and managed appropriately. All patients stated they were satisfied by the treatment offered but they dislike taking drugs continuously to treat their condition.

## Discussion

BMS is rather a rare chronic idiopathic pain condition affecting 1‰ of people, predominantly elderly women that often resist to common treatment with anti-epileptics and anti-depressants. We describe here eight cases of female patients with refractory BMS and depression who followed for more than two years. Pain was severe, daily and exaggerated during the night in all cases. Repetitive trials with anti-depressants and anti-epileptics agents commonly used in chronic pain syndromes had failed, but all responded to a combination of high dose venlafaxine and oral solution of clonazepam. Participants experienced several adverse events in the treatment initiation but after a while they tolerated it. BMS treatment remains controversial [25] yet there is little evidence that topical clonazepam helps [16]. Indeed all patients reported immediate pain improvement after clonazepam oral solution. Furthermore, increased clonazepam dose was used to treat all relapses observed successfully. As in previous reports clonazepam was used in combination of oral topical and systematic administration [26]. We used clonazepam mouthwash as reported by others [27]. Clonazepam’s mechanism of action in BMS stays unspecified. The drug binds to the peripheral GABA_A_ receptors that have been identified within the tongue nerve fibers of rats [28] and enhances GABA inhibitory effects that may control pain pathways. Additionally, clonazepam promotes brain stem serotonergic descending pain inhibition by binding to the central GABA_A_ receptors located within the brainstem [29]. Because of this dual-site action of clonazepam (peripheral and central) the drug was given both systematically and locally in our patients. Clonazepam is a long acting benzodiazepine agonist with low risk of abuse, often used for in short-acting benzodiazepine withdrawal. However, there are observations suggesting the existence of its abuse [30]. No case of clonazepam abuse was observed in our study.

Venlafaxine, a serotonin-norepinephrine reuptake inhibitor (NSRI) that controls equally depression and chronic pain [31, 32] was selected to combine clonazepam. Venlafaxine inhibits serotonin and norepinephrine reuptake leading to enhanced descending inhibition of centrally sensitized pain and has been used to treat pain conditions comorbid with depression [33]. Whether BMS, a chronic pain condition, leads to mood disturbances or in the contrary, established mental conditions might predispose an individual to symptoms related with BMS remains unanswered. Repeated observations confirm however that chronic exposure to either pain or stress can guide to maladaptive hormonal and neuronal modulations that can result in chronic pain and a wide spectrum of stress-related disorders including anxiety and depression [34], by affecting neuropeptide neurotransmission and signaling within several brain structures including the mesolimbic dopamine system [35].

Other studies showed that the majority of BMS patients present with several additional unexplained extra-oral comorbidities, indicating that various medical disciplines should be involved in the BMS diagnostic process and that BMS may be classified as a complex somatoform disorder rather than a neuropathic pain entity [36].

This study carries several limitations. It is an uncontrolled observational study with a small number of patients, thus placebo biases cannot be excluded. In a meta-analysis of placebo-controlled trials for BMS, treatment with placebos produced a response that was 72% as large as the response to active drugs [37]. These high placebo response rates documented in this review pose a significant challenge for the design of future controlled studies evaluating therapies for BMS. The aim of our study however, was to explore the potential efficacy of a combination of two pharmaceutical agents in a very selective group of BMS patients. Future controlled large scale studies are needed to provide good evidence of the suggested treatment benefits. Some patients included in this study may have not the typical clinical characteristics of BMS, e.g. pain during the night and unilateral burning, but this was the patients’ clinical picture we observed. Patients did not undergo an intraoral quantitative sensory test that has been shown to be abnormal in recent reports indicating significant loss of thermal function but not mechanical function in BMS patients, supporting the hypothesis that BMS may be a probable neuropathic pain condition [38].

BMS is often misdiagnosed and pain physicians or headache specialists should be aware of the condition. Notably, brain and facial MRI and facial nerve conduction studies showed no significant findings. Secondary burning sensation due to drugs (e.g. angiotensin converting enzyme inhibitors, dopaminergic agonists) or thyroid hormone dysregulation should be ruled. BMS has high psychiatric comorbidity but can occur in the absence of psychiatric diagnosis. Patients with atypical chronic pain syndromes must be considered as potential candidates for under-diagnosed depression (major) and suicidal thoughts. Other common BMS comorbidities include PD that should be treated accordantly. Thus, BMS management requires multidisciplinary management.

## Conclusions

Refractory BMS deserves bottomless psychiatric evaluation and management when current available treatments fail. Paraclinical investigation including brain imaging and peripheral facial nerve conduction evaluation may be needed. Controlled, large-scale trials with high dose NSRIs in combination with mouthwash and systemic clonazepam are required to establish this treatment that might help to improve pain control in this rare and severely disable pain condition.

## Abbreviations

ALA: Alpha-lipoic acid
BMS: Burning Mouth Syndrome
CI: Confidential intervals
CRP: C-Reactive Protein
HAM-D: Hamilton rating scale for Depression
ICHD-III beta: International Classification for Headache Disorders
NSRIs: Noradrenaline and serotonin re-uptake inhibitors
PD: Parkinson disease
SD: Standard deviation
SSRIs: Selective serotonin re-uptake inhibitors
TRPV1: Transient Receptor Potential cation channel subfamily V member 1 (capsaicin receptor)
